# CD34-positive circulating cells quantification during follow-up in myeloproliferative neoplasms

**DOI:** 10.1007/s00277-026-06755-1

**Published:** 2026-01-16

**Authors:** Marine Demoy, Maxence Bauvais, Agathe Vely, Thomas Nicol, Jérémie Riou, Hortense Le Jeanne, Aurélien Bauduin, Hubert Rambaud, Françoise Boyer, Franck Geneviève, Agathe Boussaroque, Marie-Christine Copin, Valérie Ugo, Corentin Orvain, Damien Luque Paz

**Affiliations:** 1 Univ Angers, Nantes Université, CHU Angers, Inserm, CNRS, CRCI2NA, F-49000 Angers, France; 2 CHU Angers, Laboratoire d’Hématologie, Angers, France; 3 Methodology and Biostatistics Department, Delegation to Clinical Research and Innovation, Angers University Hospital, Angers, France; 4 CHU Angers, Service Des Maladies du Sang, Angers, France; 5 Service de Pathologie, Université d’Angers, CHU d’Angers, Angers, France; 6 Fédération Hospitalo-Universitaire ‘Grand-Ouest Acute Leukemia’ (FHU-GOAL), Angers, France

**Keywords:** CD34-positive cell, Myeloproliferative neoplasms, Myelofibrosis

## Abstract

Polycythemia vera (PV) and Essential thrombcythemia (ET) are myeloproliferative neoplasms (MPNs) that can progress to secondary myelofibrosis, a complication associated with increased mortality. Circulating CD34-positive cells at diagnosis can be used to distinguish primary myelofibrosis from other MPNs with a threshold of 10/µL. In this study, we evaluate the interest of serial CD34-positive cell quantifications during the follow-up of MPNs. We retrospectively include 180 patients with MPN (90 ET, 55 PV and 35 myelofibrosis) with at least two measurements of circulating CD34-positive cells. CD34-positive cell count showed an AUC of 0.901 for the diagnosis of post-ET/PV myelofibrosis, with a sensitivity of 54.8%, a specificity of 99.5%, a PPV of 89.5% and a NPV of 96.3% for the threshold of 15 cells/µL. The decreased sensibility could be explained by an impact of cytoreductive treatments. Then we focused on primary and secondary myelofibrosis (MF) and found that patients achieving partial or complete response (per IWG-MRT criteria) had lower CD34-positive cell levels. Finally, CD34-positive cell levels had a prognostic impact on overall survival in MF, a count ≥ 100/µL is predictive of a higher risk of death (HR = 2.9 [1.5–5.9]), independently of DIPSS classification. In conclusion, monitoring of circulating CD34-positive cells is valuable for evaluating both disease progression and prognosis in MPN patients.

**Supplementary Information:**

The online version contains supplementary material available at 10.1007/s00277-026-06755-1.

## Introduction

*BCR::ABL*-negative myeloproliferative neoplasms (MPNs) are chronic myeloid disorders that originate from the acquisition of a somatic driver mutation in one of the *JAK2*, *CALR* or *MPL* genes [1]. These disorders are characterised by the proliferation of clonal haematopoietic stem and progenitor cells, which leads to the accumulation of mature myeloid cells. Polycythemia vera (PV) and essential thrombocythemia (ET) have an indolent evolution but can progress to secondary myelofibrosis (MF) associated with an increased risk of mortality [2].

CD34 is a sialomucin that is expressed by resident haematopoietic stem and progenitor cells in the bone marrow [3]. In physiological conditions, only a very small number of CD34-positive cells circulate in the peripheral blood [4]. However, their number can increase upon reactional (e.g. aplasia recovery, G-CSF treatment…) or malignant conditions. In MPNs, a high number of circulating CD34-positive cells at the time of diagnosis can be used to efficiently distinguish primary myelofibrosis from other MPNs with a threshold of 10 or 15 circulating CD34-positive cells per microliter of peripheral blood [5–7]. Furthermore, the count of CD34-positive cells appeared to correlate with the stage of bone marrow fibrosis [8]. A recent report by *Mannelli *et al*.* described the prognostic value of quantifying CD34-positive cells at diagnosis of myelofibrosis, with a threshold of 100 cell/µL associated with an adverse prognosis [9]. Although most studies have focused on quantifying CD34-positive cells at the time of MPN diagnosis, the dynamics of this marker may be of interest. In fact, a recent report found a correlation between CD34-positive cells and spleen size response in patients with myelofibrosis who were treated with Ruxolitinib [10].

In the present study, we aimed to evaluate the value of serial CD34-positive cell quantifications during the follow-up of MPNs. First, we validated its utility in screening for secondary myelofibrosis evolution in patients with ET or PV. Then, we examined the evolution of CD34-positive cell count in patients with primary or secondary myelofibrosis and found a correlation with treatment response and an association with overall survival, independently of the usual prognostic scoring systems.

## Materials and methods

### Patients

We consecutively included 180 patients with the following inclusion criteria: patients followed at Angers University Hospital between January 1 st, 2009 and December 31 st, 2024; with PV, ET or PMF according to World Health Organization (WHO) classifications [11]; with at least 2 assays with more than one year between first and last assay; and with a bone marrow biopsy at the time of evolution to secondary myelofibrosi**s**. Clinical data including the Dynamic International Prognostic Scoring System (DIPSS) [12], type of treatment and treatment response according to IWG-MRT [13] were retrospectively collected at each sampling point. The present project has been registered by the French Data Protection Authority (Commission Nationale de l'Informatique et des Libertés, ar20-0126v0) and received a favorable assessment from the ethics committee of Angers (number 2021–003). Only data collected during routine care were used, and no patients declined participation in the research.

### Immunophenotyping analysis

Circulating CD34-positive cells were measured by flow cytometry in total peripheral blood, collected in EDTA tubes, on the analyser BD FACSLyric™ (BD Bioscience). We use a single-tube assay with fluorescent beads to determine an absolute count of viable CD45dim CD34-positive cells (BD™ Stem Cell Enumeration Kit, BD Bioscience; Stem-Kit™ CD34 HPC Enumeration kit, Beckman Coulter). Analysis was performed on BD FACSuite™ software (BD Bioscience). Viability was assessed with 7-AAD.

### Statistics

Continous variables were described as medians with range. CD34-positive cell counts between disease groups were compared for all patients with a non-parametric Mann and Whitney test with Bonferroni correction of p-values. Receiver Operating Characteristic (ROC) analysis was used to determine CD34-positive cell count performances. Prognostic association of CD34-positive cell counts was analysed with Cox multivariable models. All analyses were performed with R software (v4.2.1, R Foundation for Statistical Computing, Vienna, Austria).

## Results

### Patients’ characteristics

Among the 180 patients included, initial diagnoses were distributed as follows: 90 ET, 55 PV and 35 MF (Supplemental Figure S1). Eighty-nine were females with a median age at diagnosis of 63.5 years (range: 21.9–88.5 years). With a median follow-up of 10.4 years, we observed 5 transformations to acute myeloid leukaemia and 1 patient evolved to chronic myelomonocytic leukaemia. Among patients with PV and ET, 11 and 22 cases progressed to secondary myelofibrosis, respectively. At the time of the most recent follow-up, 54 patients had died. Characteristics for each disease group (PV, ET or primary MF) are reported in Table 1. A total of 594 quantifications of CD34-positive cells at diagnosis and during follow-up were collected and analysed for these patients, with a median of 3 (range: 2–12) quantifications per patient.

**Table 1:** Caracteristics of patients

	**ET**	**PV**	**PMF**
Patients (*n* = 180)	90	55	35
Number of CD34-positive cell quantifications	3 (2–6)	2 (2–7)	4 (2–12)
Sex M/F	38/52	30/25	23/12
Age at diagnosis (year)	62.1 (21.9–88.5)	64.1 (26.6–85.4)	68.4 (41.5–82.0)
Mutations *JAK2* *MPL* W515L/K *CALR* TN	56 1 24 9	55 0 0 0	19 2 15 1
No transformation Transformation Secondary MF MDS/AML CMML	66 22 2 0	41 11 2 1	34 NA 1 0
Median follow-up (years)	11.0 (1.8–19.5)	10.8 (2.0–19.7)	7.3 (1.4–17.8)
Death/alive status (% death)	27/63 (30.0%)	17/38 (30.9%)	20/15 (57.1%)

*M/F male/female, TN triple negative; MDS/AML myelodysplastic syndrome/acute myeloid leukemia; CMML chronic myelomonocytic leukemia*

### Evolution to secondary myelofibrosis

**Fig. 1:**
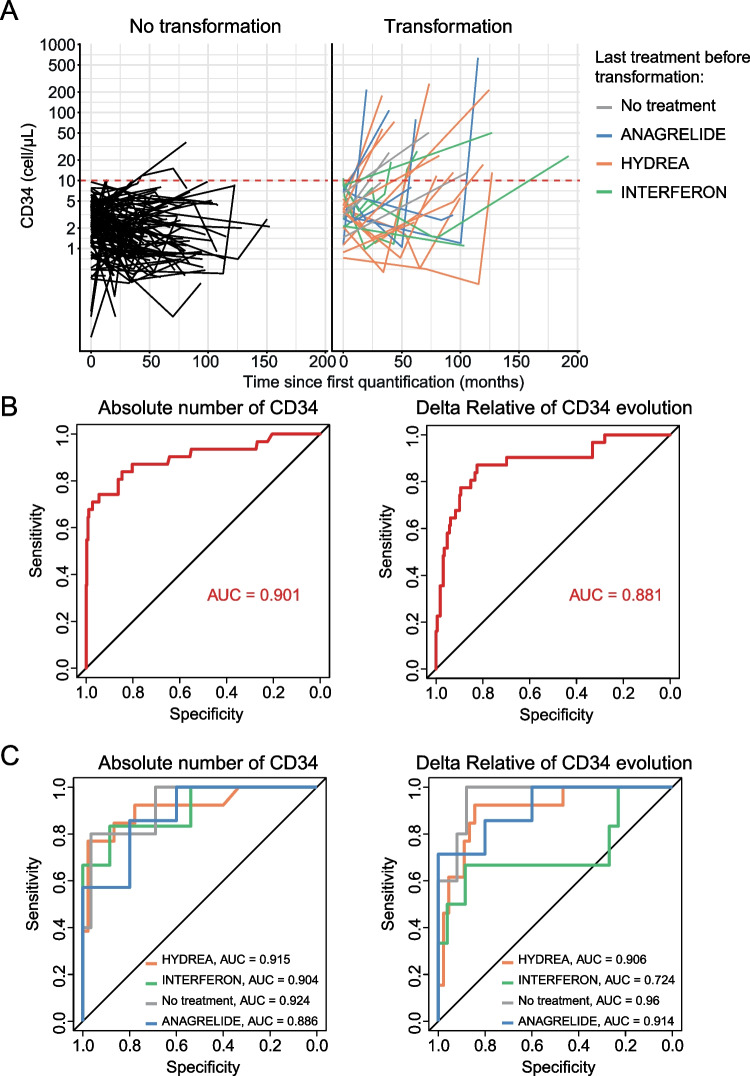
Performances of the circulating CD34-positive cell count in detecting the transformation to myelofibrosis in patients with essential thrombocytemia or polycythemia vera. A: trajectories of the quantification of CD34-positive cells over time for patients with ET or PV who did not transform during the study period (n = 104) or who evolved to myelofibrosis (n = 30). The colors represent the treatment received: no treatment (*n* = 4), anagrelide (*n* = 7), hydroxyurea (*n* = 12) or interferon (*n* = 7) B: Area under the curve (AUC) assessing the performance of the absolute number of CD34-positive cells (left) and the relative variation of CD34-positive cells (right) to detect secondary myelofibrosis in ET/PV patients. C: AUC assessing the performance of the absolute number of CD34-positive cells (left) and the relative variation of CD34-positive cells (right) to detect secondary myelofibrosis in ET/PV patients according to the treatment received

To determine whether an increased circulating CD34-positive cell count is associated with evolution to secondary myelofibrosis, we compared the absolute values and relative variations for CD34-positive cell counts in patients with PV or ET at the time of progression to myelofibrosis (*n* = 29) and patients without haematological evolution at the latest follow-up (*n* = 104). Transformation occurred with a median duration of 100 months after PV or ET diagnosis, while the median duration between diagnosis and the last follow-up in patients without haematological evolution was 72 months. The median CD34-positive cell count per µl was 19.9 (0.9–636.0) and 1.5 (0.1–36.4) for patients with and without evolution to secondary myelofibrosis, respectively (Fig. 1A). Both absolute values and relative variations of CD34-positive cell count showed good performances for the diagnosis of myelofibrosis evolution with areas under the curves of 0.901 and 0.881, respectively (Fig. 1B). Using a threshold of 10 circulating CD34-positive cells/µl, the sensibility was of 67.7%, the specificity of 98%, the positive predictive value (PPV) of 75% and the negative predictive value (NPV) of 97%. Increasing the threshold to 15 cells/µL led to a sensitivity of 54.8%, a specificity of 99.5%, a PPV of 89.5% and a NPV of 96.3%.

Among patients who transformed into secondary myelofibrosis, 4 were not receiving treatment, 9 received anagrelide, 13 received hydroxyurea and 7 received alpha-pegylated interferon at the time of CD34-positive cell quantification (Fig. 1A, Supplemental Figure S2). Among these, 3 patients without treatment, 6 with anagrelide, 10 with hydroxyurea and 5 with interferon reached the 10 CD34-positive cells/µL threshold at the time of transformation. Since treatment appeared to significantly impact CD34-positive cell counts, we calculated the area under the curve (AUC) for each treatment received (Fig. 1C). We found that the CD34-positive cell count performed better in untreated patients, particularly when using relative variation (AUC of 0.960).

Overall, we demonstrate that an increase in absolute value for circulating CD34-positive cells greater than the threshold of 10/µL in peripheral blood was highly indicative of secondary myelofibrosis in PV/ET patients during follow-up.

### CD34-positive cells follow-up in myelofibrosis

**Fig. 2:**
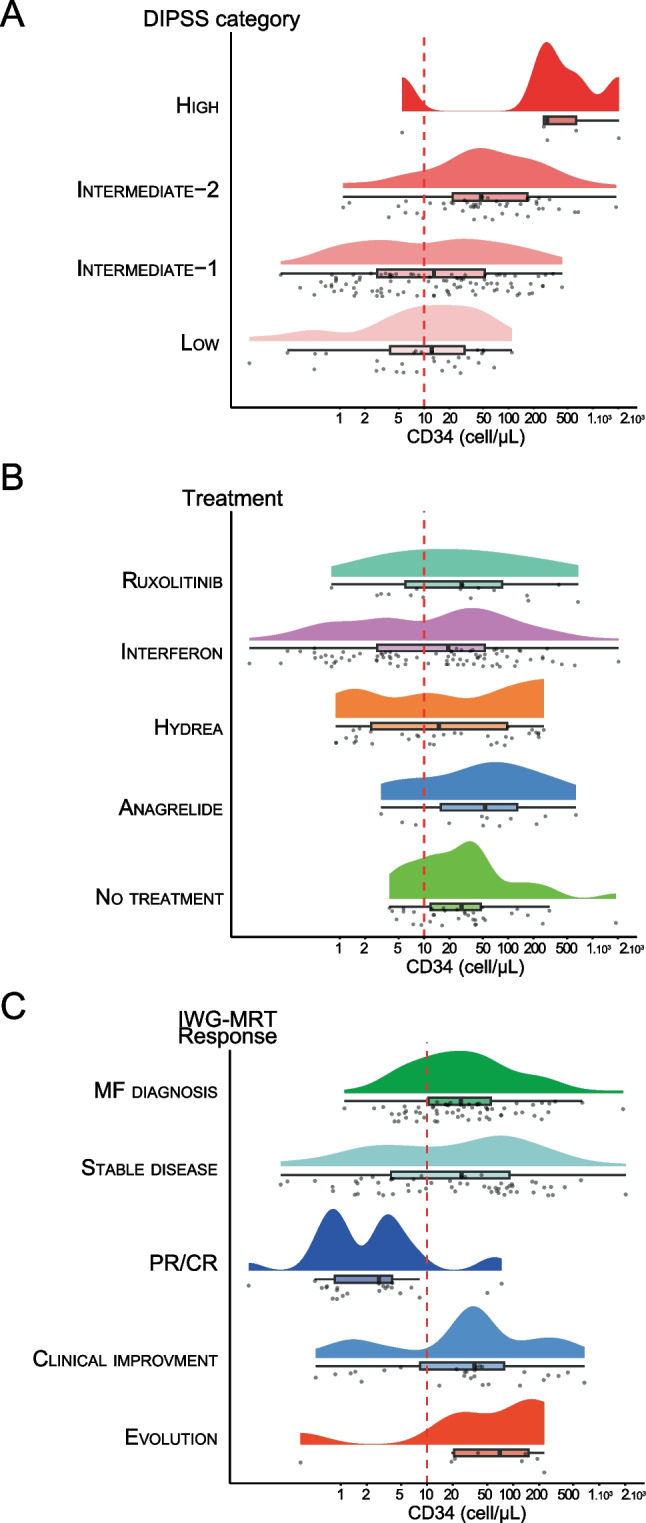
Number of circulating CD34-positive cells in primary and secondary myelofibrosis according to DIPSS group (A), treatment received (B) and IWG-MRT response (C). Rain plots aimed to compare the distribution of circulating CD34-positive cell, the boxes indicating the 75th percentile (furthest right line), median (black bold middle line), and the 25th (furthest left line) percentile of the distribution, with density curve above boxes and individual points below. *MF myelofibrosis, PR/CR partial response/complete response*

We then examined CD34-positive cell quantification during follow-up in patients with primary or secondary myelofibrosis (n = 178 measurements from n = 68 patients). Our results showed that CD34-positive cell counts were correlated with the DIPSS score calculated at each CD34-positive cells numeration (Fig. 2A). The median value of CD34-positive cells per µL was of 12.4/µl (0.1–109.9, *n* = 31) for DIPSS low, 13.1/µl (0.2–431.9, *n* = 94) for DIPSS intermediate-1, 47.7/µl (1.1–1892.6, *n* = 48) for DIPSS intermediate-2 and 289.4/µl (0.1–2032.7, *n* = 5) for DIPSS high. Statistically significant differences were observed between the DIPSS high and intermediate-1 groups (*p* = 0.0093), the DIPSS high and low groups (*p* = 0.0052), the DIPSS intermediate-2 and intermediate-1 groups (*p* = 0.0001) and the intermediate-2 and low groups (*p* = 0.0002).

Regarding treatment received, the median value of CD34-positive cells per µL was of 28.0/µl (3.9–1892.6, *n* = 34) for untreated patients, 53.1/µl (3.1–663.0, *n* = 12) for those taking anagrelide, 15.1/µl (0.9–263.7, *n* = 34) for those taking hydroxyurea, 19.3/µl (0.1–2032.7, *n* = 82) for those taking interferon and 28.0/µl (0.8–672.42, *n* = 16) for those taking ruxolitinib (Fig. 2B). However, statistical analysis did not reveal significant differences among the treatment groups.

Finally, we categorized CD34-positive cell quantifications depending on treatment response according to the IWG-MRT criteria (Fig. 2C). The median values for circulating CD34-positive cell counts were 24.8/µl (1.1–1892.6, *n* = 63) at the time of myelofibrosis diagnosis, 82.7/µl (0.34–230.0, *n* = 8) for disease progression, 25.2/µl (0.2–2032.7, *n* = 57) for stable disease, 35.5/µl (0.5–672.4, *n* = 27) for clinical improvement and 2.8 (0.1–73.5, *n* = 23) for partial or complete response. Our results showed that patients with a partial or complete response had lower values than those in all other categories (*p* = 0.0013 with disease progression, *p* < 0.0001 with diagnosis and stable disease and *p* = 0.0001 with clinical improvement). Notably, among the 14 patients who achieved a partial or complete response in our study (n = 23 measurements), the majority received interferon (8 patients), 3 received hydroxyurea, 2 received ruxolitinib, and one received anagrelide.

#### Prognostic value of CD34-positive cell counts

**Fig. 3:**
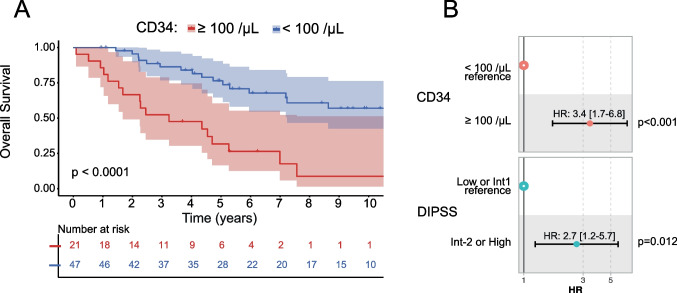
Prognostic significance of circulating CD34-positive cell quantification in patients with primary and secondary myelofibrosis. A: Kaplan–Meier model for overall survival, which separates patients with fewer than 100 CD34-positive cells per microliter (µL) at the last quantification from those with more than 100 cells per µL. B: Forest plot of the Cox model showing the hazard ratio of the association between CD34-positive cells, DIPSS stratification risk, and risk of death

Previously, a threshold of 100 CD34-positive cells per µl had been described to be associated with unfavourable prognosis in myelofibrosis patients [9]. Using this value as the threshold for the last available CD34-positive cell quantification, we were able to separate 2 prognostic groups in our cohort (*p* < 0.0001, Fig. 3A). Patients with a last quantification ≥ 100 CD34-positive cells per µl had median overall survival of 3.2 years, whereas patients with a last quantification < 100 CD34-positive cells per µl had a median overall survival of 11.6 years.

To confirm these findings regarding prognosis, we used a multivariable Cox model for overall survival considering the DIPSS at the last CD34-positive cell quantification. We found that a peripheral blood count of at least 100 CD34-positive cells per µl (hazard ratio (HR): 3.4 [1.7–6.8]), but also a DIPSS intermediate-2 or high (HR: 2.7 [1.2–5.7]) were associated with reduced overall survival (*p* < 0.001 and *p* = 0.012, respectively, Fig. 3B). As shown in Supplemental Figure S3, CD34-positive cell quantification ≥ 100/µL effectively discriminated high-risk patients within both the DIPSS Low/Intermediate-1 and Intermediate-2/High risk categories. Finally, recalculation of the DIPSS score using CD34-positive cells instead of circulating blasts yielded comparable stratification performance (C-index of 0.729 vs. 0.721 for new and classical DIPSS, respectively).

## Discussion

PMF diagnosis is based on the degree of fibrosis assessed through bone marrow biopsy analysis. Additionally, several parameters, such as increased LDH, have been incorporated into the WHO classification as minor diagnostic factors. Although determining the number of CD34-positive circulating cells is not a WHO criterion, it is useful for confirming PMF diagnosis, with a threshold of 10 or 15 cells/µL [6, 7]. Previous studies have mostly focused on quantifying CD34-positive cells at the time of diagnosis. We aimed to evaluate their characteristics during the follow-up of *BCR::ABL*-negative myeloproliferative neoplasms.

First, we demonstrate that an increase of CD34-positive cell quantification during PV and ET follow-up is highly specific of secondary myelofibrosis. The test is very accurate for excluding evolution to secondary myelofibrosis with a very good negative predictive value (97.3 to 96.3% for thresholds of 10 and 15, respectively) and had a good positive predictive value ranging from 75 to 84.5% % for thresholds of 10 and 15, respectively, making it a reliable confirmation tool for suspected secondary myelofibrosis. This could be particularly interesting for patients for whom a bone marrow biopsy is not feasible. It is worth noting that the test appeared to be less sensitive during follow-up than in historically described cohorts at the time of diagnosis [5, 7]. We could hypothesize that these false negative cases were mostly due to cytoreductive treatment. Indeed, the test had better specificity with untreated patients, and we also found that some myelofibrosis patients normalized their CD34-positive cells during treatment. Of note, four out of five patients with PV or ET who evolved to another haematological malignancy showed an increase in CD34-positive cell count at transformation (three secondary acute myeloid leukaemia and one chronic myelomonocytic leukaemia). Therefore, other haematological disorders should be ruled out when the number of circulating CD34-positive cells increases. Specifically, thrombosis occurrence was not available in this cohort, and we note that circulating CD34-positive cell quantification may also be of interest for assessing thrombotic risk, given the potential link between increased progenitor cell mobilization and vascular complications. Finally, we were unable to reclassify ET patients into early fibrotic PMF (preMF), which is associated with an increased risk of progression to overt fibrosis, due to the retrospective nature of the study, which included diagnoses from 2009 to 2024. However, in a previously well-annotated cohort, we demonstrated no significant difference in the levels of circulating CD34-positive cells between ET and preMF patients[8]**.**

Then, we found that CD34-positive cell quantification was associated to treatment response with low CD34-positive cell counts observed in primary and secondary MF patients with partial or complete response following IWG-MRT criteria. A few studies demonstrated that in patients with myelofibrosis who received ruxolitinib [10] or navtemadlin (a MDM2 inhibitor) [14] circulating CD34-positive cells decreases progressively during treatment. This decrease was also correlated with spleen size response, which is consistent with our findings in a real-world cohort including also interferon and hydroxyurea. Thus, these findings support the association between circulating CD34-positive cell levels and treatment response. However, identifying the factors that drive this association, as well as determining the relative ability of each treatment to reduce circulating CD34-positive cells, will require confirmation in a larger cohort with systematic longitudinal sampling.

Finally, we assessed the prognostic significance of circulating CD34-positive cells in patients with primary or secondary myelofibrosis. We found a correlation between CD34-positive cells quantification and DIPSS subgroup. This result is consistent with those from *Barosi *et al*.*, which found a correlation with the Lille prognostic scoring system [5]. Moreover, we found that a count of circulating CD34-positive cells ≥ 100/µL was associated with a shorter overall survival independently of DIPSS classification. In a recent study, *Mannelli *et al. developed a multiparameter flow cytometry (MFC) score using a threshold of 100 CD34-positive cells per µL and the granulocyte-to-lymphocyte SSC ratio [9]. This score enabled identification of myelofibrosis patients with reduced overall survival and improved the International Prognostic Scoring System (IPSS), Myelofibrosis Symptom Evaluation-Prognostic Model (MYSEC-PM), and Myelofibrosis International Prognostic Scoring System (MIPSS70 +) scores when used instead of the peripheral blood blast count criterion. Thus, our data confirms that quantifying circulating CD34-positive cells has a prognostic impact and can also be used during follow-up.

In conclusion, this report emphasizes the value of monitoring circulating CD34-positive cells in patients with MPNs. This rapid, standardized, inexpensive biological test enables the early detection of haematological transformation in PV and ET patients. It is also associated with treatment response and overall survival during the follow-up of myelofibrosis patients. A multicentric prospective study is necessary to confirm our results and determine the impact of circulating CD34-positive cells on the prognosis of myelofibrosis with a standardized monitoring schedule.

## Supplementary Information

Below is the link to the electronic supplementary material.

Supplementary file1 (DOCX 342 KB)

## Data Availability

The data that support the findings of this study are available on request from the corresponding author
