# Supplementary material for: CD34-positive circulating cells quantification during follow-up in myeloproliferative neoplasms
Source: Ann Hematol. 2026 Jan 16;105(1):21. doi: 10.1007/s00277-026-06755-1 (PMC12808151; doi:10.1007/s00277-026-06755-1)
Supplement: Supplementary file 1 — Supplementary file1 (DOCX 342 KB) [file 277_2026_6755_MOESM1_ESM.docx]

**Supplemental data**


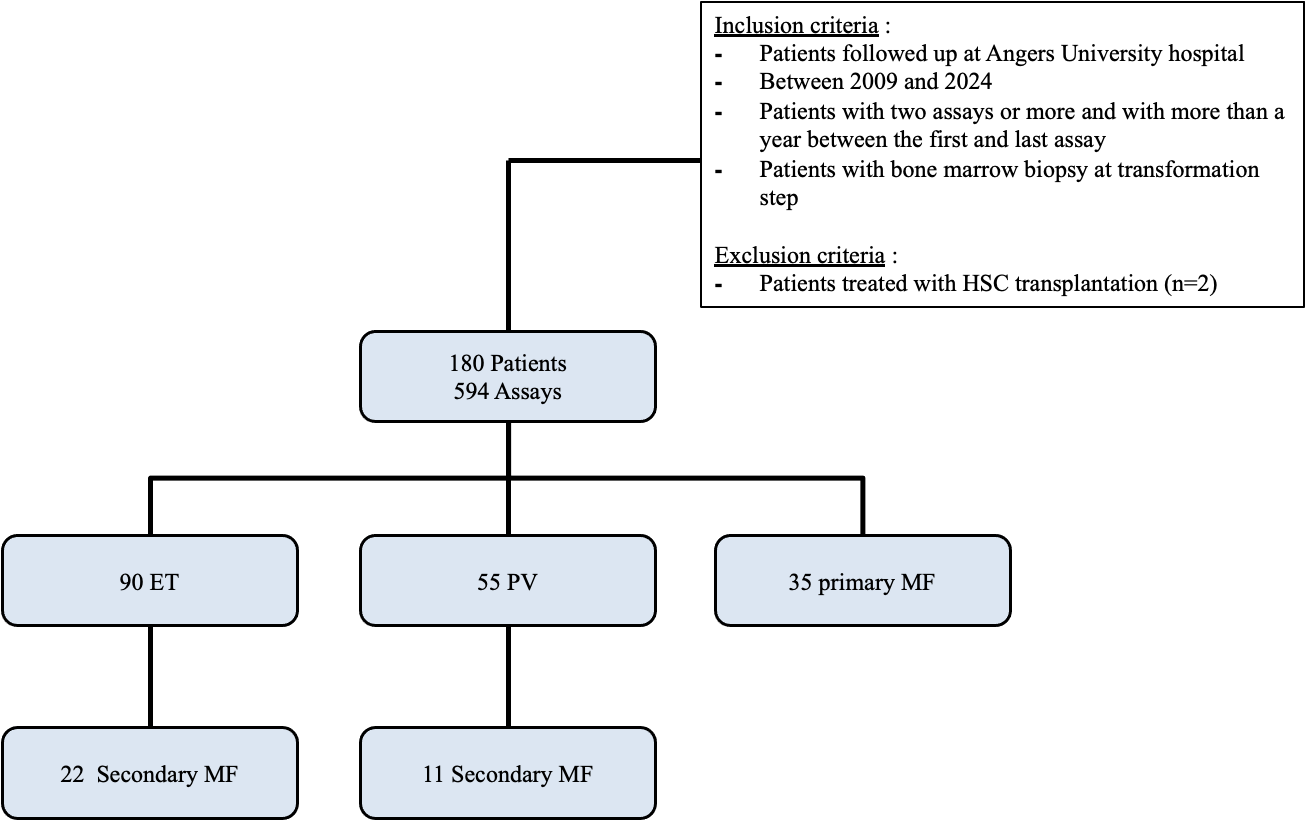


**Supplemental Figure S1**: **flow chart of the selection of patients.**

*HSC hematopoietic stem cell, ET essential thrombocytemia, PV polycythemia vera, PMF primary myelofibrosis*


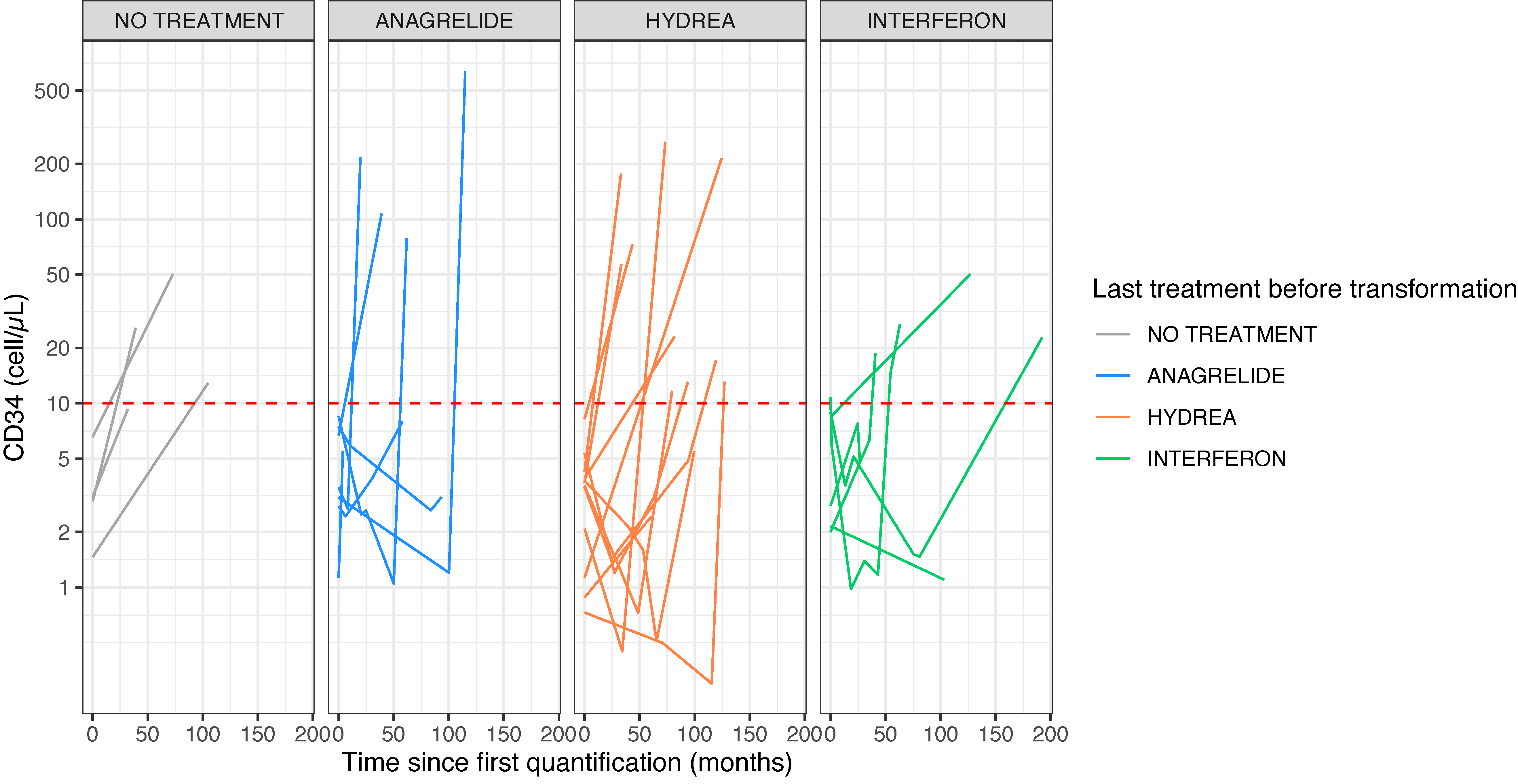


**Supplemental Figure S2**: **Dynamics of CD34-positive cell counts in patients evolving to secondary myelofibrosis, according to the treatment received at the time of transformation**

**Supplemental Figure S3. Kaplan–Meier curves illustrating the ability of CD34-positive cell quantification (≥ 100/µL) to discriminate high-risk patients within both the DIPSS Low/Intermediate-1 group (left panel) and the DIPSS Intermediate-2/High-risk group (right panel).**
